# Measuring disease activity and patient experience remotely using wearable technology and a mobile phone app: outcomes from a pilot study in Gaucher disease

**DOI:** 10.1186/s13023-019-1182-6

**Published:** 2019-09-05

**Authors:** Aimee Donald, Huseyin Cizer, Niamh Finnegan, Tanya Collin-Histed, Derralynn A. Hughes, Elin Haf Davies

**Affiliations:** 1 University of Manchester, St Marys Hospital, Manchester, UK; 2 Aparito, London, UK; 3 The Royal Free London NHS Foundation Trust, London, UK; 4 Gaucher Alliance, Dursley, UK

**Keywords:** Gaucher, Wearable technology, Mobile health

## Abstract

**Background:**

Gaucher disease is an inherited lysosomal storage disorder of which there are three subtypes. Type 1 disease has no neurological involvement and is treatable with enzyme replacement therapy. Type 2 disease results in infant death and type 3 disease is a heterogenous disorder characterised by progressive neurological decline throughout childhood and adult life. Endeavours to find a therapy to modify neurological disease are limited by a lack of meaningful clinical outcome measures which are acceptable to patients.

**Results:**

We present results from a pilot study utilising wearable technology to monitor physical activity as a surrogate of disease activity/severity paired with a mobile phone app allowing patients to complete self-reported outcome measures in the real world as opposed to the hospital environment. We demonstrate feasibility of the approach and highlight areas for development with this study of 21 patients, both children and adults.

**Conclusions:**

We illustrate, where patients engage in the methodology, a rich dataset is obtainable and useful for proactive clinical care and for clinical trial outcome development.

## Introduction

Gaucher Disease (GD) is one of the most common Lysosomal Storage Disorders resulting from deficiency of the lysosomal enzyme glucocerebrosidase, secondary to mutations in the *GBA1* gene. GD is traditionally categorised into three subtypes reflecting age of onset and involvement of the Central Nervous System (CNS); ‘Type 1’ disease is limited to systemic manifestations primarily of haematopoietic cell lines causing hepatosplenomegaly, bone marrow infiltration and osseous bone pathology but not affecting the CNS, while types 2 and 3 (nGD) involve the brain. CNS pathology in nGD primarily affects the brainstem and deep brain nuclei and progresses to involve the cerebellum and higher centres [1] resulting in a specific saccadic eye movement defect, altered muscle tone, coordination impairment, tremor and late in disease; ataxia. Patients also have varying severity bone disease, kyphosis, scoliosis, hearing impairment and other non-neurological features such as lung infiltration or cardiac disease.

Disease severity in nGD is typically described by clinicians using traditional examination techniques and, more recently, the modified Severity Scoring Tool (mSST) [2]. Although useful, these measures fail to account for the functional impact of disease on patients and only give a momentary account of function, overlooking disease fluctuations and the factors which provoke them.

Wearable technologies enable continuous monitoring of physical activity in a daily-living context, and smartphone apps can facilitate recording of Patient Reported Outcomes (PROs) and events, in real-time, to account for variable function and memory recall. Here we report the preliminary data and experience of an approach using this technology to inform our understanding of disease activity in nGD by comparing outcomes and activity within and between patients with nGD and in comparison, to a small group of patients with Type 1 Gaucher Disease.

## Results

Twenty-one patients were enrolled in the study; five patients with Type 1 Gaucher Disease age 13 yrs. – 42 yrs. (mean 24.8 yrs) and sixteen patients with nGD aged 5 yrs–48 yrs. (mean 21 yrs). Although just a convenient sample they were a relatively well age-matched but not sex matched cohort. This cohort accounts for 57% of all known UK nGD and 1.8% of the estimated UK Type 1 disease patient cohort.

Summary results are detailed in Table 1.

**Table 1:** Summary Demographics of patients enrolled in wearable activity monitoring study

	ALL (*n* = 21)	nGD (*n* = 16)	GD1 (*n* = 5)
Age (yr)	Mean: 22.3 (5–48)	Mean: 21 (5–48)	Mean: 24.8 (13–42)
Sex (M:F)	6:15	2:14	4:1
Genotype		75% L444P/L444P (others mutations include: D409H, R463C, RecNcil, E233D)	Mutations: L444P, N370S, F397S, 55bpdel, 2x large deletions
mSST	Mean: 4.76 (0–17)	Mean: 6.06 (0.5–17)	Mean: 0.6 (0–3)
6MWT (*n* = 15)		(*n* = 12); the mean distance was 391 m (median 377 m; SD 122.707)	(*n* = 3); the mean distance was 475.67 m (age range: 18-42 yrs);
6MWT Z score		−5.57 (age range: 18-42 yrs);	−3.99 (age range 6-42 yrs)

### 6 minute walk test (6MWT)

Fifteen patients completed the 6MWT; Z scores were calculated to summarise the data, using calculations by Geiger et al. [3]. The mean distance walked by nGD patients (*n* = 12) was 391 m (median 377 m; SD 122.707) and a mean z-score of − 5.57 (age range 6-42 yrs). Type 1 patients (*n* = 3); mean distance was 475.67 m (age range: 18-42 yrs); with a mean z-score of − 3.99. A difference of 1.58, BCa 95% CI[−.908, 3.805] between the two groups is identified but not significant t(14) = 1.016, *p* = .327. There was no statistical correlation between disease severity (as measured by mSST) and 6MWT (τ = −.237, 95%BCa CI [−.555, .180], *p* = .206). All but one patient showed a 6MWT score > 2 SD from the normative values irrespective of disease type.

### GaitRite/Zeno walkway

The gait analysis was undertaken as a sub study and will be reported separately.

### Wearable activity monitoring

Three patients had no ‘active days’ recorded (defined as days where > 4 epochs had recorded step data) and were thought to be non-compliant with wearing the device beyond the day of recruitment, these were all nGD patients.

Mean number of active days was 31.19 across the whole cohort (GD1 and nGD combined); median of 16 active days (SD 45.59). Patients with at least five ‘active days’ were included in more extensive analysis; *n* = 15; 5 with Type 1 disease and 10 with nGD; mean number of active days in each group was similar; 45.4 in the type 1 group and 42.3 in nGD.

Wearable device data were calculated into three different variables.

- Average Daily Maximum = ADM: The maximum number of steps per 30 min *epoch* on each active day, averaged over all active days in the month.
- Average Daily Steps = ADS: The total number of steps (from active days only) over a month, divided by the number of active days.
- Average Steps per Epoch = ADE: The total number of steps in a day divided by the number of active epochs; averaged over the number of active days in the month.

Across the whole cohort ADM was 852.1, ADS was 5293.4 and ADE was 290.0 (Table 2). When splitting GD1 and nGD data a considerable difference is noted, although not statistically significant on t-test or Mann-Whitney-U Test, with the ADS being nearly 2.5 times higher in the GD1 vs the nGD cohort. A greater difference was noted in the ADM (1537.25 vs 554.29) indicating that patients with Type 1 disease are able to perform a much higher intensity of activity in any given 30-min period.

**Table 2:** Activity parameters by disease group – Comparison by Mann Whitney U-Test

Cohort (*n* = 15)	ADM	ADS	ADE
All	852.1	5293.4	290.0
GD1 (*n* = 5)	1537.25	9805.52	489.711
nGD (*n* = 10)	554.29	3933.64	260.26
T-test	.985 *p* = .370	.885 *p* = .413	.686 *p* = 516
Mann Whitney U-Test	*p* = .768	*p* = .953	*p* = .859

Patients did not all complete the same baseline measures; correlation was performed in patients who had wearable activity monitoring of > 5 active days, 6MWT at baseline and a Gaitrite/Zeno Walkway assessment (*n* = 10). All patients included have nGD. The correlation coefficients were small between all step parameters and the other disease severity measures (see Table 3).

**Table 3:** Correlation of disease severity parameters with wearable activity monitoring results in nGD patients

Kendall’s Tau	Average Daily Maximum	Average Daily Steps	Average Steps per Epoch
6MWT			
Correlation Coefficient	−0.09	− 0.045	0
Sig. (2-tailed)	0.719	0.857	1
N	10	10	10
mSST			
Correlation Coefficient	0.068	−0.068	− 0.205
Sig. (2-tailed)	0.787	0.787	0.417
N	10	10	10
Velocity (GAITRite/Zenomat)			
Correlation Coefficient	−0.244	−0.156	−0.111
Sig. (2-tailed)	0.325	0.531	0.655
N	10	10	10

Adherence to device use was not correlated with age; however, Pearson correlation coefficients between average step counts and age; ADM and age r = −.592, *p* = .071; ADS and age r = −.593, *p* = .071 and ADE and age r = −.573, *p* = .084 is large. This suggests that older patients, in this cohort of Gaucher patients, are less active, mSST scores in patients with nGD do deteriorate over time [2] but are not directly correlated with age (some patients with nGD are more severe in childhood) and a low correlation between age and mSST was seen when measured by Pearson’s correlation r = −.338 *p* = .340.

When factoring the effects of bone disease and kyphosis, age was not correlated, however ADS and severity of bone disease were correlated; Kendalls τ = −.538; *p* = .012, and kyphosis to ADS showed a moderate correlation; Kenadalls τ = −.367 *p* = .080 although not statistically significant.

### Phone application results

Three patients reported no events and didn’t respond to any of the app PROs and were considered to be non-adherent with phone application usage; one patient did not engage with the wearable also (nGD patient), one patient had difficulty synchronising the phone app to the device and reported they had lost data (T1 patient) and the other patient felt too busy to use the app (type 1 patient).

### Event reporting

**Fig. 1:**
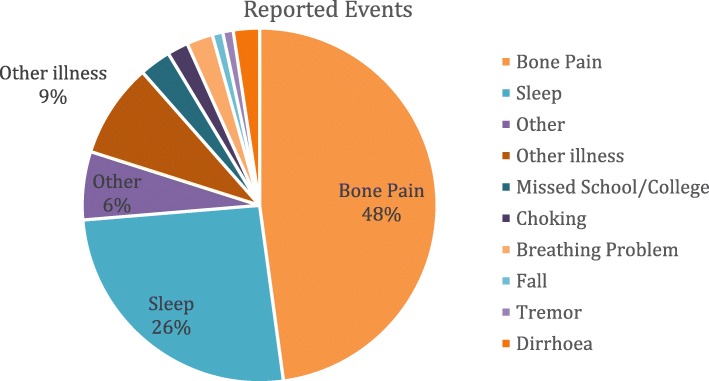
Frequency of reported events via the phone app*.* Legend: Pie chart showing reported events, colour coded by frequency as percentage and colour coded to depict bone pain, sleep, other event, other illness, missed school, choking, breathing, fall, tremor, diarrhoea

Thirteen patients; nGD *n* = 9 (56%) and GD1 *n* = 2 (40%), reported ‘events’ using the app on at least one occasion. There were 210 events reported in total, ranging from 1 to 102 per patient. The majority of events were recorded by nGD patients and the most frequently reported event was ‘bone pain’. Only two events (sleep impairments) were reported by GD1 patients. Details of the reported events are presented in Fig. 1. All events reported by patient and details of the ‘other’ events can be found in Table 4 and Table 5.

**Table 4:** Events Reported on App; Event Type and Number

Pt N:	Disease Type	No. of Events	Event type (number reported)
002	3	6	Bone Pain (1), Fall (1), Absence from education/work (1); Tremor (1); Other Illness (1); Sleep Impairment (1); Other: Sleepy and clumsy after infusion
003	3	4	Bone Pain (2); Absence from education/work (1); Other illness (1)
004	3	8	Bone Pain (2); Absence from education/work (1); Other illness (1); Sleep Impairment (4)
005	3	102	Bone Pain (38); Breathing Problems (4); Choking Episodes (4); Diarrhoea (5); Fall (1); Other Illness (10); Sleep Impairment (31); Tremor (1); Other: Anxiety/Depression (3); Headache (1); Toothache (2); Swallowing difficulty (1); General lethargy post infusion (1)
008	3	1	Absence from education/work (1)
009	3	2	Bone Pain (1); Other illness (1)
010	3	3	Bone Pain (1); Absence from education/work (2)
013	3	10	Sleep impairment (3); Other illness (2); Other (5): Sore throat, Headache, Restless legs, Abdominal Pain, Constipation
018	3	70	Bone Pain (54); Breathing Problems (1); Sleep impairment (13); Other (2): Swallowing difficulty, Knee pain.
020	1	1	Sleep impairment (1)
021	1	1	Sleep impairment (1)

**Table 5:** ‘Other’ Events reported

Other Description	Frequency
Clumsy	1
Headache	2
Swallow	2
Fatigue	1
Depression	1
Anxiety	2
Restless Legs	1
Abdominal Pain	1
Constipation	1
New co-morbid diagnosis	1

The two patients who reported the greatest number of bone pain events were both nGD patients, one patient (patient 018) with very severe bone disease requiring multiple surgical interventions, the other patient (patient 005) has relatively minimal objective evidence of bone disease but was the highest app user. The high reporting rate may, in part, reflect a differing event reporting threshold to other patients. A further factor may reflect time period of app usage. Patient 005 had a 318-day reporting period compared to a 44-day reporting period for patient 018. As such; the ratio of bone events reported was 0.12 events per day compared with 1.23 events reported per day for patient 018.

It is important to note that 7 out of the 9 nGD patients reported bone pain as an event indicating that this is a significant disease feature across the cohort, even though it might be over shadowed by other clinical manifestations.

Likewise, sleep impairments were reported by 5 out of the 9 patients a total of 49 times (26%). Patients were asked to report sleep ‘events’ via the question ‘did you sleep poorly?’, if they responded ‘yes’ they could detail the reason they attributed; the majority of responses reflected ‘restlessness’, ‘anxiety’, excessive thoughts, feeling too hot or having pain. Whether the sleep impairment was caused by bone pain specifically is not clear, but an association should be considered. The combined impact of both of these events can be seen to have significant impact on day-to-day physical activity.

Ratios of events:reporting day appear to reflect clinical findings of disease activity, however also highlight the symptomatic experience of patients which may be overlooked clinically by traditional monitoring or failure of patient recall in a clinic setting.

### Patient reported outcomes (PROs)

**Fig. 2:**
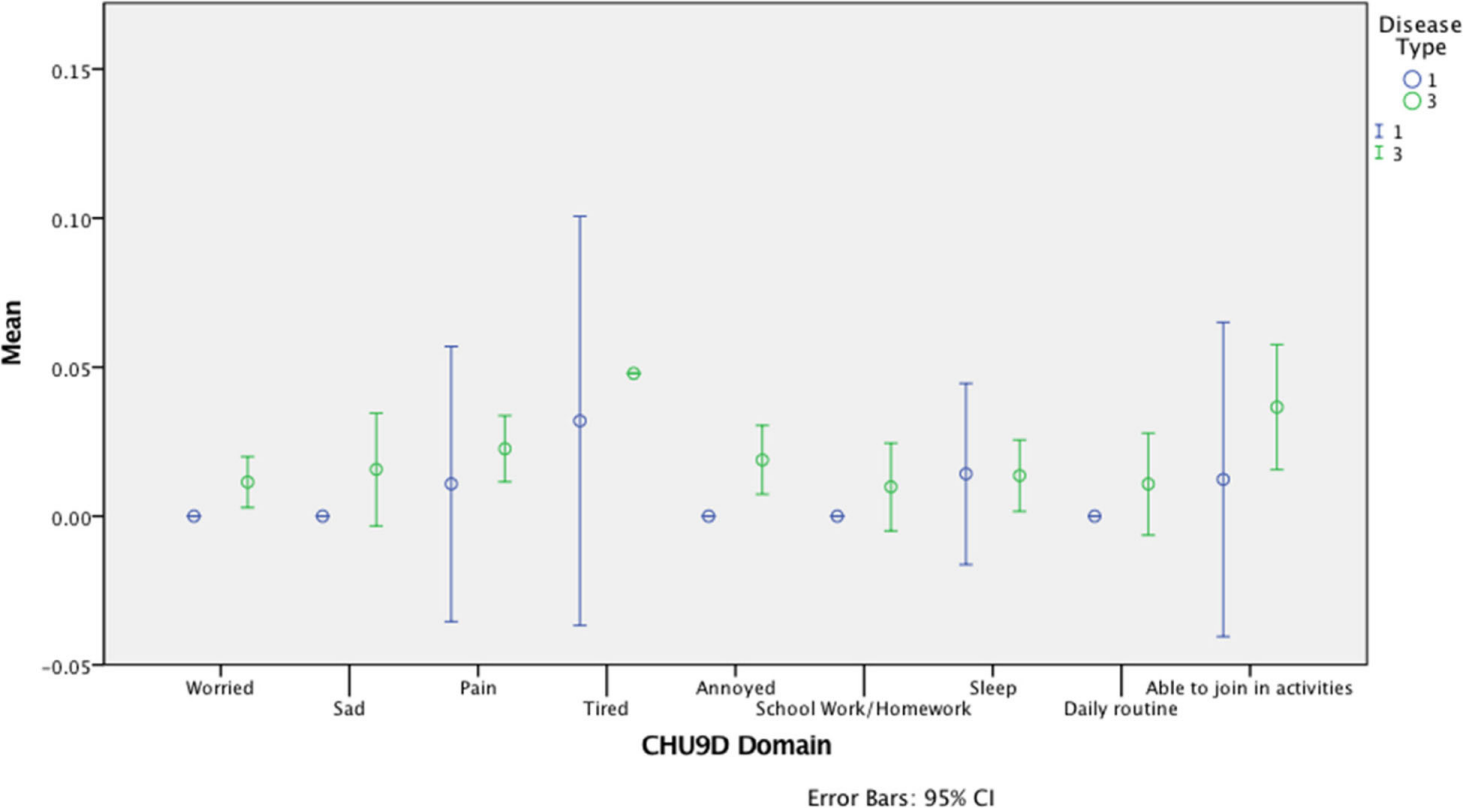
CHU9D scores by disease type. This chart shows the CHU9D domains across the x-axis and the mean scores for the domain by patient group, blue bars = type 1 disease and green bars = type 3 disease, the distribution shows the mean and 95% confidence intervals

The results from the PRO’s in the form of mean scores (in relation to the scoring reference ranges) and statistical differences between disease groups are detailed in Table 6 and Fig. 2.

**Table 6:** Patient Reported Outcomes mean scores by disease type

PRO	Number of Patients Completed		Mean Score		Independent t-test between groups	PRO ref. range
	GD 1	nGD	GD 1	nGD patients		
CHU9D*	3	10	**0.93 (SE = .016)**	**0.81 (SE = 0.025)**	**t(11) = 2.43;** ***p*** **= .033**	0.33–1 (higher score = perfect health)
PedsQL MFS*	3	10				0–100 (higher score = less fatigue)
General Fatigue			69.44 (SE 2.78)	56.25 (SE 6.37)	t(11) = 1.09; *p* = .298	
Sleep Fatigue			56.99 (SE7.35)	58.08 (SE 8.32)	t(11) = −.07; *p* = .945	
Cognitive Fatigue			69.44 (SE 15.47)	57.08 (SE 4.89)	t(11) = 1.04; *p* = .321	
Self-esteem: Rosenberg Self-worth*	2	10	**14.5 (SE = 0.5)**	**16.5 (SE = 0.5)**	**t(10) = −1.70;** ***p*** **= .119**	0–30 (< 15 = Low self-esteem)
Perceived Stress Scale*	2	10	**6.5 (SE 4.5)**	**22.7 (SE 6.17)**	**t(10) = − 3.38;** ***p*** **= .007**	0–40 (higher score = greater perceived stress)
REM Sleep Behaviour Disorder Questionnaire	5	11	1.4 (SE .872)	5.64 (SE .877)	t(14) = −2.94; ***p*** **= .011**	> 5 = problematic sleep
Pittsburgh Sleep Quality Index	5	10	3.8 (SE 2.17)	8.8 (SE 1.76)	t(13) = −1.90; *p* = .079	> 5 = problematic sleep
Sleep Disturbance Scale for Children	0	3	NA	72.67	NA	Total T-Score > 70 pathological* (Subdomains scored identically)

Note 1: *Data from the ‘Global self-worth’ scale was not analysed as no patients under the age of fourteen completed this.*

*PROs completed on the Aparito phone application.

The CHU9D showed a statistically significant difference between disease groups, nGD patients reporting overall lower health-related quality of life. Figure 2 shows that fatigue (“tired”), as a CHU9D measured domain, showed the highest scores in both patient groups, but a greater range in the patients with Type 1 Gaucher disease.

**Fig. 3:**
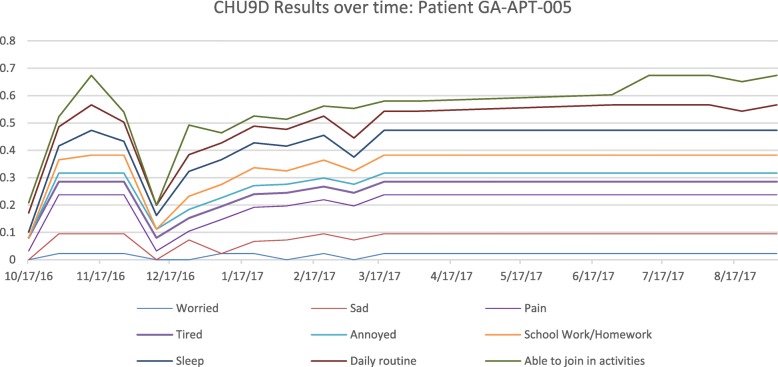
Patient 005 CHU9D Responses Over Time. This chart shows a single patient (005) with time (date) on the x-axis and the y-axis shows the score at each time point for each domain of the CHU9D, reflected in a line graph (per domain (colour coded))

Correlation between domain responses is demonstrated when we look at the most active PRO responder; patient 005 who showed consistency between responses measured in the scale as illustrated in Fig. 3.

Although not statistically significant; patients with nGD generally reported slightly higher levels of fatigue as measured by the MFS. Domains of fatigue; ‘general’, ‘sleep’ and ‘cognitive’ were consistent in suggesting poor sleep patterns across most patients with no single domain dominating deficits.

The sleep specific PROs showed pathological scores in the nGD group which may reflect underlying neuropathology and contribute to fatigue scores. Problematic sleep as detected by the REM Sleep Behaviour Disorder Questionnaire (RSBDQ) was detected in patients with nGD but not in patients with Type 1 Gaucher disease, while the Pittsburgh Sleep Quality Index did show a difference between disease groups, but not quite statistical significance. This perhaps suggests the nature of sleep disturbance in nGD is specific (as measured by the RSBDQ), further analysis of this will follow as a study extension.

Results from the sleep-specific PROs were correlated with PedsQL MFS and with ‘sleep disturbance’ event reporting through the phone app. Table 7 details the mean scores for these parameters; correlation was identified between the self-reporting questionnaires and the number of sleep events reported by patients.

**Table 7:** Sleep assessment tool correlations

	RSBDSQ	PSQI	Mean Total MFS	MFS Sleep Sub-score	Total Sleep Events Reported
Total Mean	4.31	7.1333	55.86	52.8	0.16
Type 1 Mean	1.4	3.8	65.28	56.94	.5
nGD Mean	5.64	8.8	52.33	51.25	5.2
Pearsons R (Total Sleep Events reported)	.660 (*n* = 14)	.735 (*n* = 13)	−1.66 (*n* = 12)	−.729 (*n* = 11)	
p	.010	.004	0.721	0.011	

**Fig. 4:**
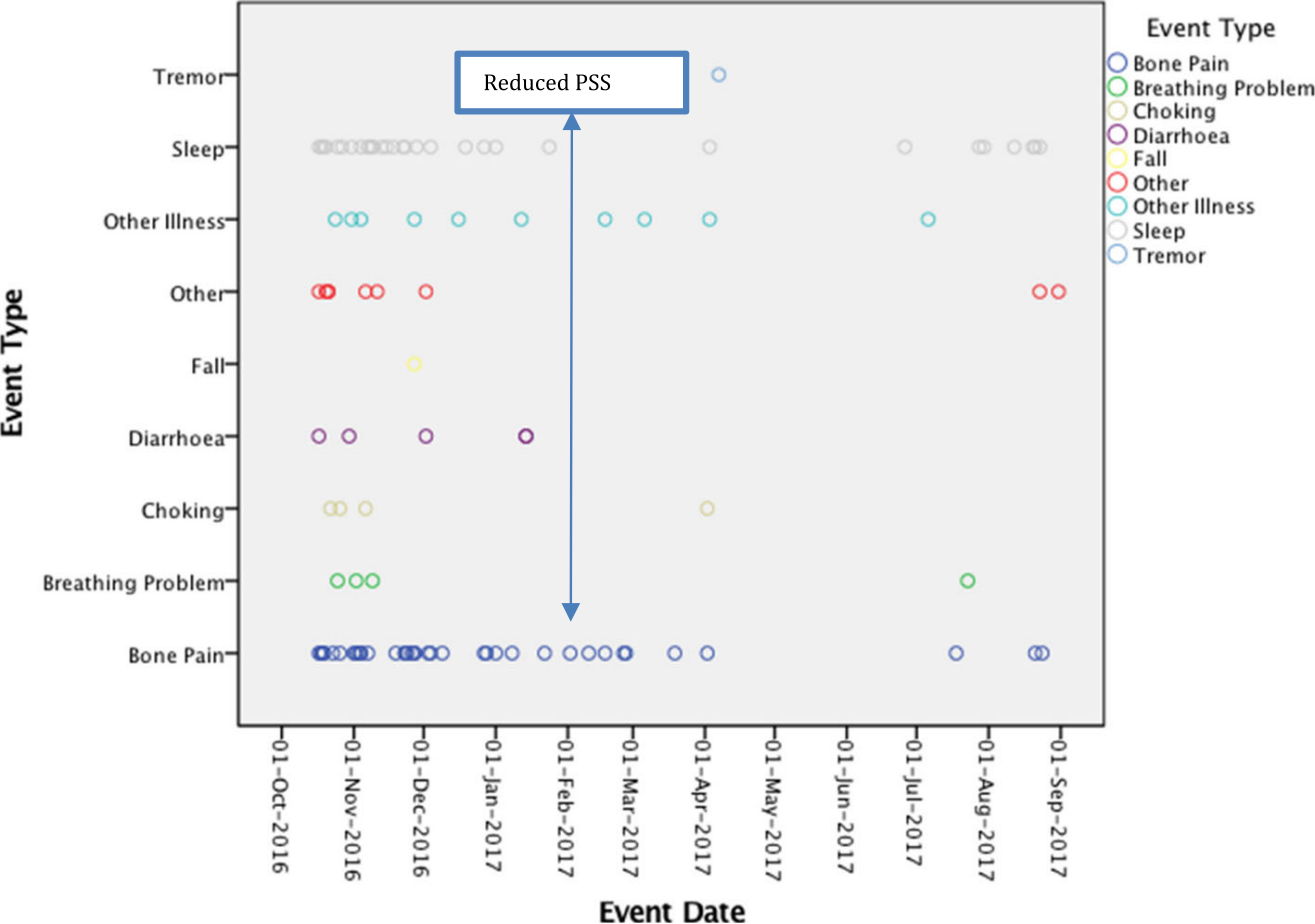
Patient 005 Reported Events. This graph records the reported events (y-axis) patient 005 recorded by date (x-axis) with a highlight of the date at which the perceived stress score (pss) value reduced

Perceived stress was also significantly higher in patients with nGD than patients with type 1 Gaucher disease (although only 2 patients with T1 disease responded to the questionnaire at baseline). Two patients showed specific fluctuations in PSS over time; patient 05 had a decline in stress score (PSS) in February 2017 which was correlated with a reduction in the number of events recorded during this period (Fig. 4). Patient 14 who also showed a change over time, did not record any events at all to be able to determine the nature of the change in perceived stress.

### Correlation of activity/PROs/events

**Fig. 5:**
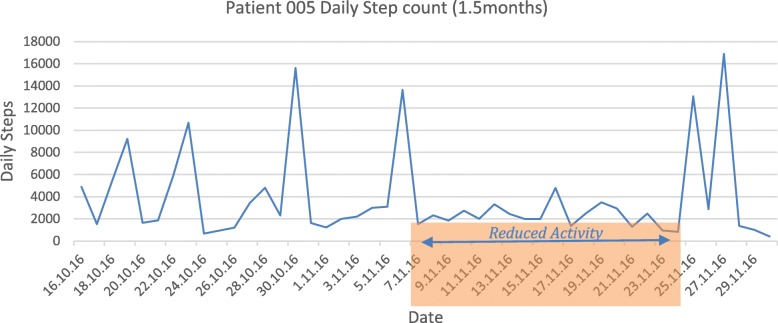
Patient 005 step count over time. This figure shows dates on the x-axis and the daily step count (y-axis) for patient 005, this line graph shows the change in activity over a specific period of time - highlighted on the graph

Patient 005 was the most active user of both the wearable device and the app and is used to illustrate the utility of correlating step data, event and PROs in combination. At the time of peak difficulties in “joining in activities” and “performing daily routines” as reported in the CHU9D (November 2016) a decline in step count is also observed (Fig. 5). When correlated with number and type of reported ‘events’ via the app at this time, the patient reports poor sleep due to increasing anxiety symptoms, demonstrating the possible enrichment and interpretation of both the PRO and activity data by using the real-time event reporting, and giving objective illustration of the overall picture for clinical staff to see.

## Discussion

The wearable device variables (Average Daily Maximum = ADM; Average Daily Steps = ADS; Average Steps per Epoch = ADE) measured in this study calculated important differences between the nGD and GD1 cohort. The ADS was nearly 2.5 times higher in the GD1 cohort than the nGD cohort, with an even greater difference noted in the ADM (1537.29 vs 554.29), indicating that patients with GD1 are able to perform a much higher intensity of activity in any given 30-min period. High intensity activity requires not only physical strength but also coordination. The presence of ataxia, tremor etc. might therefore impact on the patient’s ability to partake in high intensity activity regardless of physical strength alone. Although some correlation between ADS and bone disease was seen, a more detailed study with greater patient numbers would be required to determine the nature of the difference in step counts seen, it is likely that the combined effects of bone disease and neurology are contributory. As this study has highlighted, bone pain in this cohort of nGD patients has a greater functional impact on activity and quality of life than perhaps previously recognised. The gait analysis data offered by the GAITRite and Zeno Walkway although show a difference between disease groups, with identifiable gait parameter deficits suggestive of a neurological basis of impairment, are not adequately defined to enable correlation with the step data. Poor sleep and fatigue are also likely to be contributing factors reducing total amount of physical activity each day. This observation demonstrates the value of combining the PRO and event reporting in the app with the wearable device and the importance of monitoring more general aspects of quality of life in multisystem rare neurodegenerative disease.

When comparing ADS values in this cohort to other studies it is noted that nGD patients were much less active (mean ADS of 3933.64) compared to those reported in cohorts of patients with Multiple Sclerosis (mea*n* = 5478) [4] but similar to the mean ADS reported in patients with Pompe disease (unassisted ambulation); ADS = 3408 [5].

The phone app patient reporting aspect of the study showed broader utility than first imagined. Not only were the PRO assessments considered to be easier for patients to interact with and more likely to give a reliable reflection of experience, the event reporting offered patients the opportunity to highlight functional aspects of their disease experienced perhaps previously overlooked. nGD studies historically have focussed on neurological symptoms and here patients reported fatigue and bone pain as significant symptoms of disease.

The sleep impairments reported by patients correlated well with formal validated measures of sleep and although don’t offer detail on the nature of impairment do provide an opportunity to examine the impact of sleep to activity. This specific group of patients have an extremely low frequency of seizures but in other cohorts the effect of a seizure on activity and sleep, for example, could be identified and then the effect of therapeutic intervention recorded in a relatively objective way in real time. Utilising a wearable device to measure sleep parameters in future larger studies is planned.

This pilot study primarily served to assess the feasibility of using such technology in this patient group. Long term adherence in using the wearable device, and consistent engagement with the app impacted on the analysis. It wasn’t entirely clear whether events weren’t reported because they weren’t experienced, the patient didn’t want to report them or didn’t fully understand how to do so on the app. Patient adherence to use of the technology appeared to be impacted by three main factors;

- Technical failures
- Training and on-going support
- Patients capability to cope / being easily overwhelmed.

Some technical failures and limitations impacted on the patient’s ability to achieve high engagement and adherence; especially download and Bluetooth synchronisation of the app across different mobile handsets. This was compounded by the fact that patients needed a lot of training and on-going support with the technology which was not always possible to provide quickly (with a small study team and patients distributed throughout the UK at several different centres), along with what seemed to be a low threshold for getting overwhelmed with instructions. A complete analysis of the relationship of engagement to IQ wasn’t possible as formal IQ testing wasn’t undertaken as part of the study, however, using clinical judgement and historical cognitive assessments a relationship appeared present between those patients with greater cognitive deficits and lower engagement. Such patients have very specific intellectual impairments and strive for independence. Although they were encouraged to seek support in undertaking study activities (using the app and device), often they lacked motivation to seek help in this regard. This was less relevant in the paediatric age group where the technology was managed by parents. For younger children however, devices were often too big for the wrist, were lost easily or damaged more easily and required frequent replacement.

Some patients also reported that the phone app offered no feedback regarding data obtained; many young adults wanted to be able to track their own activity and parents felt a symptom diary would be useful for recalling events during hospital appointments. Such features had intentionally been excluded from the app interface so as to limit the exposure of unfiltered raw data to patients however future deployment should address this request.

Since this pilot study there have been significant changes to the underlying technology, with the aim to simplify and improve the user experience. Based on the feedback of the wearable device specifically, another device has also been selected which meets much of the informal feedback offered by patients. Ability for doctors to log on to system during clinic to review all data is also now possible.

The technology however is beneficial to both clinical patient care and research. It makes participation in care and research accessible to patients and offers timely feedback to clinical and academic teams using methodologies increasingly familiar to both parties in a technologically advancing society.

## Conclusions

This pilot dataset has demonstrated both the feasibility and utility of this approach to disease assessment which addresses many of the unmet needs in this patient group. An expanded study with consideration of the practical and logistic limitations identified is required to adopt this into both clinical and research environments with subsequent expansion across disease areas.

## Methods

Patients were recruited from specialist UK centres and through the UK Gaucher Association. Patients with a genetic and biochemical diagnosis of Gaucher Disease over the age of 5 years, ambulant and who were able to comply with at least three of the study procedures were approached for participation.

Patient demographics and disease state are presented in Table 8.

**Table 8:** Wearable technology: Patient demographics, disease characteristics & study engagement

Pt No.	Sex	Age	Disease Type	Genotype	Treatment	Baseline mSST	Bone Disease	Kyphosis/Scoliosis	Other Co-morbidity	Ambulation	No. of active days (> 4 epochs)	Average no. of active epochs per active day	No. of Events	Total PROs Completed
1	F	29	3	L444P/L444P	ERT	14	Moderate	Mild		Fully Ambulant	0	0	0	2
2	F	21	3	L444P/L444P	ERT + SRT	7	Severe	Moderate		Reduced ambulation - acute injury	1	9	6	8
3	F	19	3	L444P/L444P	ERT	5	Moderate	Moderate		Fully Ambulant	35	12.91	4	11
4	F	22	3	L444P/L444P	ERT	2.5	Mild	Mild		Fully Ambulant	5	16	8	5
5	F	23	3	L444P/L444P	ERT	2.5	Mild	Mild		Fully Ambulant	184	20.39	102	36
6	F	18	3	L444P/L444P	ERT	11	Mild - Moderate	Mild		Fully Ambulant	0	0	0	1
7	F	15	3	L444P/L444P	ERT	3	Mild	Mild		Fully Ambulant	15	19.93	0	9
8	F	16	3	L444P/E233D	ERT + Ambroxol	17	Mild	Moderate		Requiring support - Neurology	47	15.51	1	8
9	F	22	3	L444P/L444P	ERT	2	Moderate	Mild		Fully Ambulant	23	13.56	2	4
10	F	8	3	L444P/D409H	ERT	0.5	Mild		Juvenile Arthritis	Fully Ambulant	2	13	3	0
11	M	7	3	L444P/L444P	ERT + SRT	1.5	Mild	Mild		Fully Ambulant	1	11	0	0
12	F	5	3	L444P/L444P	ERT	1.5	Mild	Mild		Fully Ambulant	10	18.9	0	0
13	F	34	3	L444P/L444P	ERT	4	Mild - Moderate	Mild	Rheumatoid Arthritis	Fully Ambulant	56	9.34	11	10
14	F	36	1	L444P/N370S	ERT	0	Mild	Mild		Fully Ambulant	103	16.05	0	12
15	M	42	1	N370S/ Other Deletion	ERT	0	Mild	None		Fully Ambulant	77	17.1	0	0
16	F	27	3	L444P/L444P	HSCT	12.5	Moderate	Surgical Correction		Limited by pain	0	0	0	0
17	F	31	3	R463C/D409H	ERT	5	Mild	Surgical Correction		Limited by pain	7	16.25	1	4
18	M	48	3	R463C/RecNcil	ERT	8	Severe	Surgical Correction		Requiring support - Pain	18	14.22	70	0
19	M	18	1	F397S/Other Deletion	ERT	3	Mild - Moderate	Moderate		Fully Ambulant	16	15.44	0	3
20	M	13	1	N370S/55bpdel	ERT	0	Mild	None		Fully Ambulant	14	17.36	1	0
21	M	15	1	N370S/55bpdel	ERT	0	Mild	None		Fully Ambulant	6	20.17	1	4

Baseline clinical assessments included a neurological examination, the mSST, 6 Minute Walk Test (6MWT) and GAITIRite or Zeno Walkway gait analysis. The GAITRite/Zeno Walkway gait systems are transportable walkways embedded with pressure sensors which detect footfall in realtime allowing measurement of stance, gait velocity, balance and weight distribution amongst a range of more complex gait parameters.

Although the mSST was designed specifically to evaluate the neurological manifestations of patients with nGD, mSST scores derived from the neurological examination of type 1 Gaucher patients were also generated for the purposes of comparison. The domains evaluated in the mSST are standard neurological disease features which collectively are relevant to nGD but are not exclusive to the disorder, e.g. seizures accounts for one domain but seizures have multiple causes. In addition, the kyphosis domain scored in the mSST also occurs in non-neurological disease (type 1 patients) secondary to bone disease of the vertebrae and may impact on activity.

The 6MWT was completed on a 25-m track following a standardised trial procedure. Z scores were calculated to summarise the data, using reference ranges for age and gender generated by Geiger et al. [3]; the z-score was calculated by subtracting patient scores for the reference values for age and sex [3] and divided by the standard deviation of those reference values.

All consenting patients downloaded the Aparito application (App) to their own or their parents’ mobile phone. This app was paired with a 3D accelerometer device (Million pedometer) to be worn on the wrist and patients were encouraged to keep this in situ at all times for the duration of the study; minimum of 2 weeks, maximum of 12 months. The accelerometer device captured data in 30 min epochs and calculated the number of steps taken for that 30-min period. The paired app pushed out Patient Reported Outcomes (PROs) and Quality of Life (QoL) scales at pre-set intervals ranging from fortnightly to every 2 months. Table 9 lists the PROs and the frequency in which they were sent out.

**Table 9:** Patient Reported Outcomes and Quality of Life Scale details and scheduling

Patient Reported Outcomes	Domains	Function	Age Group	Timing Schedule (Days)
CHU 9D (Child Health Utility 9D)	9	Health-related Quality of Life	Paediatric but validated for use in adolescents and used previously in adults [6, 7]	14
PedsQL™ Multidimensional Fatigue Scale	General Fatigue Sleep Fatigue Cognitive Fatigue	Fatigue Measure	Age specific scales	30
Perceived Stress Scale	10	Perception of ‘stress’	Adolescents & Adults	30
Global Self Worth (subscale from ‘Self-perception Profile for Children’) [8]	6	Self-esteem	Children aged 8 yr-14 yrs	60
Rosenberg Self Esteem [9]	10	Self-esteem	Adults	60
REM Sleep Behaviour Disorder Questionnaire [10]	10	Detection of REM Sleep Behaviour Disorder	Adults	Baseline
Pittsburgh Sleep Quality Index [11]	7	Identification of problematic sleep	Adults	Baseline
Sleep Disturbance Scale for Children [12]	6	Identification of type of sleep disorder in children	Children	Baseline

Patients could also record visits to health care professionals, other ‘events’ e.g. falls, seizures etc. and they were encouraged to offer detail of sleep quality. Sleep was evaluated on paper-based questionnaires using validated tools at baseline and report episodes of poor sleep through the phone app in real-time.

## Data Availability

The datasets used and/or analysed during the current study are available from the corresponding author on reasonable request.
